# Adipose Tissue and Lipid Dysregulation Contributions to Cachexia, Sarcopenia and Muscle Function

**DOI:** 10.1002/jcsm.70299

**Published:** 2026-04-27

**Authors:** Joseph D. Abraham, Stephan von Haehling, Stefan D. Anker

**Affiliations:** ^1^ The Heart and Vascular Center and The Carl and Edyth Lindner Research Center The Christ Hospital Cincinnati Ohio USA; ^2^ Department of Cardiology and Pneumology University of Göttingen Medical Center Göttingen Germany; ^3^ German Centre for Cardiovascular Research (DZHK), Partner Site Göttingen Göttingen Germany; ^4^ Berlin Institute of Health‐Center for Regenerative Therapies (BCRT) Charité ‐ Universitätsmedizin Berlin Berlin Germany; ^5^ Deutsches Herzzentrum der Charité, Department of Cardiology (Campus Virchow) Charité ‐ Universitätsmedizin Berlin Berlin Germany; ^6^ German Centre for Cardiovascular Research (DZHK), Partner Site Berlin Berlin Germany

The role of adipose tissue in cardiovascular disease has been increasingly understood as having a strong, central role in systemic metabolic and inflammatory regulation. Adipose tissue is more than a passive energy reservoir; it functionally acts as an endocrine organ itself that can influence cardiometabolic risks, skeletal muscle integrity and progression toward sarcopenia and cachexia. In contemporary clinical practice, obesity and muscle wasting together with underlying chronic illness such as heart failure or chronic kidney disease frequently coexist as comorbid disease processes.

The core concept is that lipid metabolism within adipose tissue plays a critical role in inter‐organ signaling. In a comprehensive review, Jang and Choi demonstrate that dysregulated lipid handling within adipose tissue directly contributes to skeletal muscle dysfunction through inflammation, oxidative stress, insulin resistance and mitochondrial impairment. They emphasize that lipid accumulation itself is not inherently pathological, but the metabolism through lipid turnover and intracellular localization can have negative roles. They further describe mechanisms by which obesity disrupts physiological signaling pathways and, in the context of cachexia, highlight remodeling of white adipose tissue toward thermogenic, brown adipose tissue [1].

Expanding that pure weight or body size alone does not define risk, but that body composition is clinically richer, Amin and colleagues argue that models based on body mass index (BMI) are missing a part of the picture. In a large, observational cohort, they show that individuals with similar BMI but differing regional body fat distributions have significantly different fracture risks. Accordingly, they propose a novel measure of partial body fat percentage [2]. This supports a broader principle that BMI is a crude tool, and further characteristics, such as regional adiposity, may be more clinically and biologically meaningful. The limitations and improvement on our characterizing tools also need to be standardized, as methodological heterogeneity in sarcopenia research can complicate further interpretation across studies [3].

Wan and colleagues extend the body composition argument into systemic organ disease. In patients with both obesity and biopsy‐proven non‐alcoholic fatty liver disease (NAFLD), a higher CT‐derived visceral fat index was significantly associated with disease progression [4]. These findings reinforce that regional lipid distribution, especially visceral, highly metabolic lipid, has roles in disease progression.

Kim and colleagues provide insights into muscle quality rather than solely muscle quantity. Analysing 1440 patients with CT imaging data from the mid‐thigh, they found that reduced myosteatosis was associated with greater muscle strength and better functional performance, independent of gender differences or diabetes mellitus status. This opens myosteatosis as a potential therapeutic target for sarcopenia prevention [5]. Muscle mass preservation and functional recovery have also emerged as a target in other disease states [6]. Importantly, this supports that preserved muscle bulk could still have lipid infiltration contributing to a poorer muscle quality with consequences on functional capacity and frailty.

The concept of altered body composition does translate to symptom burden. Anker and colleagues propose a ‘muscle hypothesis’ of shortness of breath in cachexia. They argue that in cachexia, dyspnoea is not adequately explained from cardiac or pulmonary failure but is additionally explained by skeletal and respiratory muscle wasting, cytokine‐driven catabolism and hyperactivation of the metabo‐ergoreflex [7]. The compelling clinical translation of the adipose‐muscle interactions is suggestive that once lipid dysregulation is present and muscle function becomes dysfunctional, dyspnoea symptoms can have contributions outside of haemodynamics from a sinister negative feedback loop of worsening myopathy.

Hameed and colleagues found no meaningful improvement in age‐adjusted mortality from heart failure with cachexia from 2004 through 2020 [8]. In parallel, Waqas and colleagues, analyzing more than 5.5 million hospitalizations, showed frailty is associated with increased risk in hospitalized heart failure with reduced ejection fraction (HFrEF) patients. Frailty was common and strongly associated with inpatient mortality, shock, mechanical ventilation, among other morbidities [9]. Together, these studies suggest that wasting syndromes are integral to disease severity rather than being a secondary complication.

Moving from body composition to lipid biology, Khan and colleagues highlight the role of lipoprotein(a) as a genetically determined cardiovascular risk factor. Approximately 20% of the global population has elevated lipoprotein(a) levels, which have strong links as a cardiovascular disease risk factor [10]. Importantly, adiposity alone does not capture risk. Lipid biology matters through inherited atherogenic pathways that independently amplify risk.

Clinically, we have many therapeutic tools to address lipids, but we must make sure they reach patients. While lipoprotein(a) screening is recommended, screening is heavily underutilized [10]. Ngina and colleagues studied more than 5.1 million individuals and found that more frequent lipid monitoring was associated with progressively lower incidence of major adverse cardiovascular events and improved event‐free survival [11]. Risk modification includes addressing dyslipidaemia over time with escalations in lipid management. This has been reinforced with Rehman and colleagues' national analysis that demonstrated dyslipidaemia as a significant contributing factor to stroke‐related mortality [12].

Collectively, these studies support a more integrated clinical framework. Adipose dysfunction drives abnormal lipid metabolism, visceral fat accumulation, myosteatosis, declining muscle quality and eventually sarcopenia and frailty. These processes contribute to worsening symptom burden, increased inpatient risk and higher cardiovascular mortality. Accordingly, risk stratification should extend beyond traditional metrics to include novel body composition markers, muscle quality and complete lipid and genetic profiling. This type of approach may allow earlier identification and therapeutic intervention to prevent major adverse cardiovascular events [13].

The broader importance of this subject is that it unifies prevention and prognosis. Many of our cardiovascular disease risk factors are, more aptly, just risk factors. Obesity, frailty, cachexia, dyslipidaemia, BMI and other disease‐ and risk‐related factors are closely interrelated and may adversely interact, thereby affecting lipid and muscle health, metabolic processes and mortality.

## Data Availability

Data sharing not applicable to this article as no datasets were generated or analyzed during the current study.
